# Effect of insulin on small intestinal transit in normal mice is independent of blood glucose level

**DOI:** 10.1186/1471-2210-6-4

**Published:** 2006-02-01

**Authors:** Peddyreddy Murali Krishna Reddy, Steven Aibor Dkhar, Ramaswamy Subramanian

**Affiliations:** 1 Department of Pharmacology, JIPMER, Pondicherry-605006, India; 2 Department of Pharmacology, AVMC, Pondicherry-605006, India

## Abstract

**Background:**

Insulin is the drug of choice in the management of diabetes mellitus (DM). About 76 % of diabetic patients suffer from gastrointestinal (GI) disorders. Therapy of DM with insulin primarily involves lowering of elevated blood glucose levels. Hence, on any organ in addition to insulin's effect, hypoglycaemic effect also prevails. A systematic study exploring the effect of insulin on small intestinal transit in normal laboratory animals is lacking. Hence, in the present study, the possible effect of insulin with or without associated hypoglycaemia on small intestinal transit in normal mice was examined.

**Results:**

Insulin in all the doses tested (2 μ, 2 m and 2 U/kg) elicited a significant acceleration of SIT. The lower doses of insulin (2 μ and 2 m U/kg) produced significant acceleration of SIT and were associated with normal blood glucose levels. However, the highest dose of insulin (2 U/kg) produced an acceleration of SIT that was associated with significant fall in blood glucose levels. Further, the 2 m and 2 U doses of insulin significantly elevated serum insulin and C-peptide levels.

**Conclusion:**

Insulin at the lowest dose produced an acceleratory effect on SIT that was independent of blood glucose and serum insulin levels in normal mice.

## Background

Diabetes Mellitus (DM) is a major chronic metabolic disorder affecting a large portion of population world wide [1]. The diagnosis as well as the progression/remission of DM are usually based on evaluation of biochemical parameters *viz*., blood glucose, serum insulin and C-peptide levels [2]. Assessment of serum insulin level in newly diagnosed DM is helpful in deciding about the type of oral hypoglycaemic agent to be prescribed [3]. Since the therapy is for life time, the probability of development of complications is more [4]. Complications involving the enteric neuropathy causes considerable morbidity in patients with long standing insulin-dependent diabetes with poor glucose control and peripheral neuropathy [5,6]. About 76% of DM patients suffer from gastrointestinal symptoms [7]. These symptoms include gastroparesis, abdominal pain, diarrhoea, fecal incontinence and constipation [8]. Motility studies of small intestine in diabetes are not extensive and available reports are contradictory to one another [6,9]. In addition, the small intestine is generally considered as the main site of drug absorption [10]. Patients with diabetic diarrhoea will have less time for absorption of drugs taken orally [4].

Insulin is the drug of choice in controlling hyperglycaemic state in Type 1 and sometimes for Type 2 diabetes [4]. Insulin has been reported to increase the gastric emptying through hypoglycaemic effect [11]. Different segments of GI tract are regulated by independent or different mechanisms [10]. It is well known that insulin therapy primarily involves reduction in blood glucose levels [4]. Hence on any organ, in addition to hypoglycaemic effect, insulin's effect may also prevails. A systematic study exploring the effect of insulin on small intestinal transit in normal laboratory animals is lacking. Therefore, we propose to study the effect of insulin on small intestinal transit by using varied doses in normal mice. This may provide an experimental evidence for the unexplained relief of gastrointestinal complications in DM when treated with insulin.

## Results

### Effect of insulin administration on small intestinal transit, blood glucose, serum insulin and C-peptide levels

Insulin administration at lower doses (2 μ and 2 m U/kg) did not alter blood glucose levels significantly but produced a significant acceleration of SIT (P < 0.01) (Table 1). However, the higher dose (2 U/kg) produced an acceleration of SIT that was associated with a profound fall in blood glucose levels (P < 0.01). Insulin at all the three doses elevated serum insulin and C-peptide levels but the statistical significance was achieved only at 2 m or 2 U dose.

**Table 1:** Effect of exogenous insulin administration on blood glucose (BG), serum insulin, C-peptide levels and small intestinal transit (SIT) in normal mice

**Treatment Insulin (U/kg;sc)^a^**	Blood glucose levels			**%SIT^e^**	**Serum insulin levels^f ^**(μU/ml)	**Serum C-peptide levels^g ^**(ng/ml)
				
	**Initial (mg/dL)^b^**	**Final (mg/dL)^c^**	**% Change^d^**			
Vehicle	70.66 ± 5.97	71.66 ± 5.93	101.96 ± 5.40	47.47 ± 3.22	32.62 ± 6.83	0.250 ± 0.01
2 μ	188.8 ± 29.73	198.8 ± 14.68	110.9 ± 10.65	68.43 ± 3.59**	55.18 ± 2.9	0.271 ± 0.01
2 m	128.16 ± 8.07	123.33 ± 13.62	94.88 ± 6.43	61.53 ± 4.42*	144.74 ± 13.43**	0.395 ± 0.02**
2	135.66 ± 8.76	53.66 ± 3.38	41.27 ± 5.60**	73.08 ± 2.18**	178.66 ± 12.23**	0.470 ± 0.01**

Each value represents the mean ± SEM (n = 6)

*P < 0.05; **P < 0.01 as compared with vehicle treated group

^a ^Insulin administered (s.c) 50 min before blood glucose/SIT estimation

^b ^Initial blood glucose levels were estimated before insulin/vehicle administration

^c ^Final blood glucose levels were estimated 50 min after insulin/vehicle administration (time coinciding with SIT determination)

^d ^% change (in blood glucose levels) obtained from any alteration in final BG value from initial BG level of each animal

^e ^Values are % SIT considering the total length of the intestine as 100, SIT was determined 50 min after insulin administration

^f ^Independent blood samples were collected at the time coinciding with SIT measurement for the measurement of serum insulin levels

^g ^Independent blood samples were collected at the time coinciding with SIT measurement for the measurement of C-peptide levels

### Effect of insulin administration on % change in SIT and blood glucose levels

Insulin administration with lower doses (2 μ and 2 m U/kg) produced significant acceleration of SIT and were associated with normal blood glucose levels. However the higher dose of insulin (2 U/kg) produced an acceleration of SIT that was associated with a significant fall in blood glucose levels (P < 0.01) (Fig 2).

**Figure 1:**
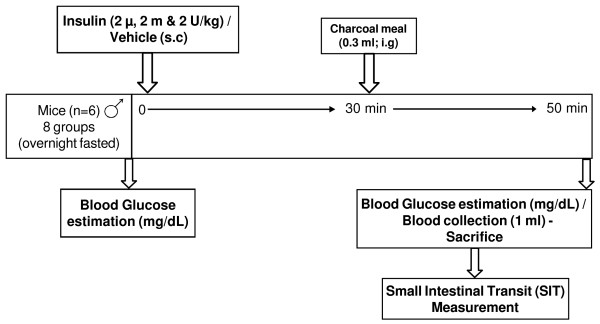
Experimental design carried out to study the effect of insulin administration on small intestinal transit (SIT) in normal mice.

**Figure 2:**
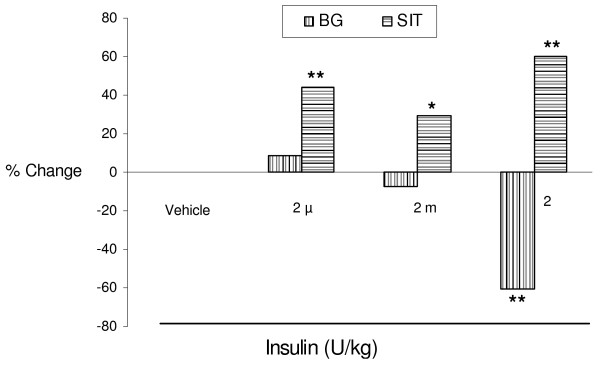
Effect of insulin administration on % change in small intestinal transit (SIT)and blood glucose (BG) levels in normal mice. Values are expressed in terms of % change (increase or decrease) when compared with respective vehicle treated group. *P < 0.05; **P < 0.01 when compared with respective vehicle treated group.

### Effect of insulin administration on % change in SIT and serum insulin level

**Figure 3:**
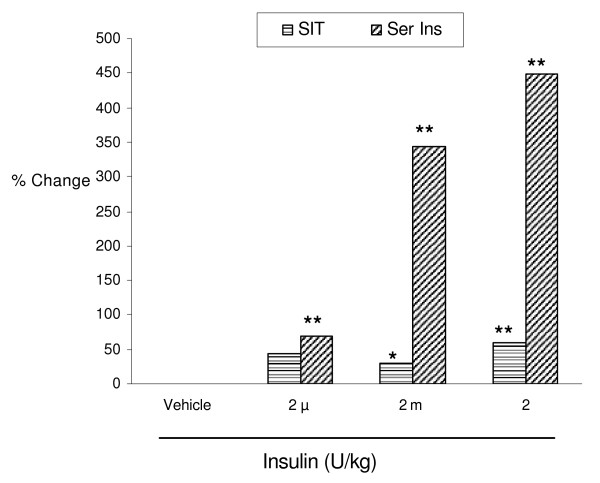
Effect of insulin administration on % change in small intestinaltransit (SIT) and serum insulin (Ser Ins) levels in normal mice. Values are expressed in terms of % change when compared with respective vehicle treated group. *P < 0.05; **P < 0.01 when compared with respective vehicle treated group.

In contrast to glycaemic states, with all the doses of insulin administration a positive relationship between the changes in SIT and insulinemic states was recorded (Fig 3). While insulin in all the doses tested produced similar degree of acceleration of SIT, the effect on serum insulin levels was noticed to be dose related.

## Discussion

Our study with insulin administration demonstrates a novel finding that lower doses of insulin significantly accelerated small intestinal transit without altering blood glucose levels (Table 1). Generally, the parenteral administration of insulin produces maximal fall in blood glucose levels within 20–30 min [4]. Therefore, to study the effect of insulin and associated fall in blood glucose levels on intestinal transit, the charcoal meal was administered 30 min after insulin administration. We used varied doses of insulin to avoid or sustain the hypoglycaemic effect, and to explore an inherent effect of insulin on SIT. Bulatao and Carlson [14] observed the hypoglycaemic effect of insulin in dogs was associated with an increase in the height and frequency of gastric contractions and gastric tonus. These effects depend on blood glucose levels [15]. Later studies indicated the acceleration of gastric emptying [11] and other parts of intestine [16] were due to the hypoglycaemic effect of insulin. In our experiment, blood glucose levels were not affected with lower doses of insulin (2 μ and 2 m U/kg) but produced a significant acceleration of SIT. However, the higher dose of insulin (2 U/kg) produced an acceleration of SIT that was associated with a significant fall in blood glucose levels (P < 0.01) (Fig 2). This may indicate that an indirect role for hypoglycaemic state in acceleratory effect of insulin on SIT at the high dose. Hence, we suggest that blood glucose levels do not play a role at least in lower doses of insulin in accelerating SIT.

Insulin acts indirectly on stomach through vagus nerve to increase the gastric emptying [17]. In fact, it was emphasized that insulin injection could be used as a challenge to test the functional integrity of vagus fibers to the stomach. At one time, insulin effect on gastrointestinal (GI) motility was recommended as a model to compare prokinetic drugs on GI motility, which is comparable to metoclopramide [18,19]. In addition, it was also reported that insulin may have a permissive role in maintaining basal pattern motility in the sheep intestine [20]. Some contrasting reports are also available about production of depressant effect on GI motility by releasing norepinephrine in rats [21,22] and some attributed this inhibitory effect due to hyperpolarization of smooth muscle [23] or to the phenol content of commercial insulin [24]. Moreover, these observations were recorded at higher concentrations of insulin [14,18,20].

To rule out the influence of endogenous insulin on SIT, we felt it is necessary to measure the serum insulin levels. We observed that serum insulin levels were not altered with the lowest dose of insulin (2 μU/kg). But with the higher doses of insulin (2 m and 2 U/kg), the serum insulin levels were significantly elevated (P < 0.01) (Fig 3). These findings indicate that exogenously administered insulin is solely responsible for significant acceleration of SIT, but with the higher doses of insulin elevated endogenous insulin levels produced cannot be ruled out in acceleration of SIT. The elevated serum insulin levels observed with higher doses of insulin might be due to insulin administration itself or a reflex release of endogenous insulin from the pancreas. Garvey et al [25] reported about an improvement of endogenous insulin secretion after exogenous insulin injection. There is also a clinical evidence indicating that intensive insulin treatment in Type 2 diabetes improve β-cell function [26]. These reports also support a view that exogenous insulin administration may have a reflex action over the secretion of insulin from pancreas in healthy animals.

To further elucidate the role of endogenous insulin levels on SIT with higher doses of insulin, we evaluated C-peptide of insulin levels. C-peptide is released in equimolar ratio with insulin from islet cells and is not extracted by liver [27]. Hence, the C-peptide measurement reflects the absolute status of endogenous insulin production. Normally the C-peptide level is expected to come down by supplementation of insulin. Surprisingly, we noticed that C-peptide levels in serum were significantly elevated with higher doses of insulin administration (P < 0.01) (2 m and 2 U/kg) (Table 1). In this animal model, the above observation further confirms the view that higher doses of exogenously administered insulin reflexly stimulate the synthesis of insulin which resulted in release of C-peptide. These findings strengthen the view that the lowest dose of insulin (2 μ/U/kg) inherently accelerated SIT without the involvement of hypoglycaemia and endogenous insulin levels in normal mice.

Our subsidiary experiment about *in vitro *effect of insulin on mouse ileal longitudinal smooth muscle indicated that all the dose ranges of insulin (2, 20, 200 μ, 2, 20, 200 m and 2 U) tested, a marked increase in tonus of the muscle was produced. The available literature indicates that insulin produces a transitory inhibition followed by marked increase in tonus in the gastrointestinal smooth muscle [23]. Our results also indicate a similar phenomenon that is an increase in tonus but without transitory inhibition at all the ranges of insulin doses. This finding suggest a convincing evidence for the myogenic effect of insulin on ileal smooth muscle.

In this study, an excellent intestinal motor response to insulin administration was obtained with the lower doses of insulin (2 μ and 2 m U/kg) without the influence the blood glucose levels. It is known that muscle contraction is dependent upon its ability to utilize glucose [28]. Thus, the ability of intestinal muscle to utilize glucose might have been facilitated by insulin administration resulting in the observed acceleration of SIT. However, a centrally mediated hypoglycaemic effect may not be ruled out for the acceleratory effect on intestine with the higher dose of insulin (2 U/kg) [23].

## Conclusion

The lowest dose of insulin (2 μU/kg) which accelerated the small intestinal transit in healthy animals is without any significant changes in the blood glucose, endogenous insulin and C-peptide levels. It may be solely attributed to the inherent effect of insulin which is independent of its hypoglycaemic response. Further studies are required in diabetic animals to explore the acceleratory effect of insulin in relieving constipation.

## Methods

### Animals

Randomly bred normal adult Swiss albino male mice, weighing between 20–25 g were obtained from central animal house, JIPMER, Pondicherry. One week before the study, animals were housed at departmental animal house in polypropylene cages under standard laboratory conditions. Later, animals were divided into eight groups (n = 6). Experiments were performed during the day (9 am to 6 pm). The experimental protocol was approved by JIPMER institutional animal ethics committee.

### Drugs and chemicals

Insulin injection I.P (purified bovine insulin, 40 U/ml; Knoll Pharmaceuticals Ltd, Aslai; India), Wood charcoal from SD Fine Chemicals, Boisar, Gum Acacia IP from Hikasu Chemicals, Mumbai, were used. Radio-immuno Assay kit for insulin estimation was obtained from BRIT, BARC, Mumbai, India, Radio-immuno Assay kit for C-peptide of insulin estimation was procured from Diagnostic Systems Laboratories, Inc, Texas, USA. Insulin injection was diluted with normal saline solution for obtaining a suitable strength.

### Measurement of blood glucose (BG) Levels

The Blood glucose level was measured by placing a drop of blood obtained by venipuncture of tail, over an appropriate glucostix, read by Advantage Glucometer (Boerhinger Mannheim Corporation, Indianapolis, USA) and expressed as %, change in the glucose level considering the initial value of that animal as 100. The blood glucose measurement is based on bioamperometry, the enzyme glucose dehydrogenase present in the test strip converts the glucose in the drop of blood to gluconolactone. This reaction creates a harmless electrical current that the meter interprets as blood sugar. This estimation was done 10 min before insulin administration and 2–3 min before sacrificing the animal for measuring small intestinal transit (Fig 1).

### Measurement of small intestinal transit (SIT)

The SIT was determined by identifying leading front of intragastrically administered marker in small intestine of an animal [12]. Charcoal meal marker was freshly prepared by dispersing 10 g of wood charcoal in 5% gum Acacia mucilage in purified water. Each mouse received 0.3 ml of this suspension intragastrically (i.g) using metallic oral cannula. After 20 min animals were sacrificed by intravenous administration of sodium pentobarbital (100 mg/kg), abdomen was cut open, the leading front of marker was identified in the small intestine and tied immediately to avoid movement of marker. The entire length of small intestine was isolated by cutting at pyloric and ileocaecal ends. The distance travelled by charcoal meal and the total length of the intestine were measured in cm(s). The SIT was expressed as percentage (%) of the distance travelled by the charcoal meal to length of the intestine. This was done in animals 50 min after insulin administration (Fig 1).

### Measurement of serum insulin and C-peptide of insulin

Serum insulin or C-peptide was measured by collecting 1 ml of blood by rupturing orbital plexus and the serum was separated by centrifuging at 1000 g and stored at -20°C till the assay of insulin was performed. Serum insulin or C-peptide was estimated by radio-immuno assay (RIA) technique [13] on a Wallac OV-20101 Wizard Automatic Gamma Counter, Turku, Finland. This measurement was done independently but at the time coinciding with SIT measurement (Fig 1). Different groups of animals were used for estimating serum insulin or C-peptide. The inter-assay coefficient of variation of Radio-immuno assay kit for insulin estimation (RIAK-1) was reported as less than 15 %, and intra-assay coefficient of variation was reported as less than 10 %. The inter-assay coefficient of variation of C-peptide RIA kit (DSL-7000) of 5 duplicates was reported as 2.4 %, and intra-assay coefficient of variation of 10 duplicates reported as 3.3 %.

### Administration of Insulin

Each group of overnight fasted mice was administered insulin subcutaneously (s.c) 2 μ, 2 m (milli) and 2 Units/ kg or vehicle (Fig 1). Blood glucose level was recorded before insulin administration. Charcoal meal was administered (i.g) 30 min after insulin administration and SIT was determined after 50 min.

Four separate groups of animals were treated similarly without SIT measurement but one ml of blood was collected through orbital plexus at the time coinciding with SIT measurement for the measurement of serum insulin or C-peptide levels.

### Statistical analysis

Results are expressed as mean ± SEM and analysed statistically using ANOVA followed by Dunnett's multiple comparisons test. P < 0.05 was considered as statistically significant.

## Abbreviations

BG: Blood glucose; DM: Diabetes Mellitus; GI: Gastrointestinal; i.g: Intragastric; RIA: Radioimmunoassay; s.c: Subcutaneous; SEM: Standard error of mean; SIT: small intestinal transit

## Authors' contributions

PMKR involved in conception, design, experiments, acquisition of data, analysis and preparation the manuscript. SAD verified the study design and suggested corrections in the manuscript. SR designed the study, analysed the data and revised the manuscript. All authors read and approved the final manuscript.
